# Plant‐Derived Exosome‐Like Nanoparticles: Innovative Nanomedicine for Therapeutic Applications

**DOI:** 10.1002/fsn3.70974

**Published:** 2025-09-22

**Authors:** Zihan Gao, Jiajia Li, Wenhui Yang, Yuning Gu, Wenrong Xu, Zhaofeng Liang, Jiajia Jiang

**Affiliations:** ^1^ Aoyang Institute of Cancer Affiliated Aoyang Hospital of Jiangsu University Suzhou P.R. China; ^2^ Department of Laboratory Medicine, School of Medicine Jiangsu University Zhenjiang P.R. China

**Keywords:** anti‐inflammatory, antioxidant, antitumor, delivery, plant nanoparticles, therapeutics

## Abstract

Plant‐derived exosome‐like nanoparticles (PELNs) have demonstrated substantial potential and promising prospects in the realms of disease prevention and therapy. Composed of a diverse array of bioactive constituents and secondary metabolites, they are poised to exert significant influence in anti‐inflammatory, antitumor, and antioxidant activities. Furthermore, their inherent nanomaterial properties coupled with excellent biocompatibility render PELNs natural candidates for drug delivery systems, enhancing bioavailability. This paper mainly reviews the biogenesis, isolation, and characterization of PELNs, then endeavors to synthesize the current body of knowledge on them and emphasize their potential therapeutic applications. We focus on key findings regarding anti‐inflammatory, antitumor, and antioxidant effects and discuss how these insights can guide the design of next‐generation nanomedicines.

## Graphical abstract

This graphical abstract outlines the extraction process of plant‐derived exosome‐like nanoparticles (PELNs) and their potential applications in the treatment of various diseases. Enriched with miRNAs and proteins, PELNs can be used for targeted therapy of inflammatory diseases, novel applications in cancer therapy, the unique role of PELNs in antioxidant stress, treatment of liver diseases, treatment of skin diseases, the unique role of PELNs in antimicrobial infections, potential applications of PELNs in neurological diseases, and PELNs as a platform for delivering bioactive molecules.

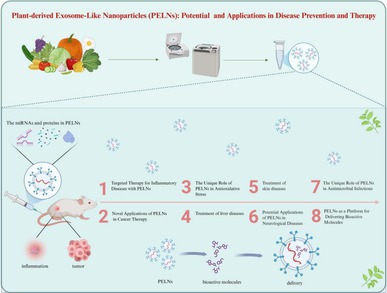

## 1 Introduction

In the recent period, the swift progress in the domains of nanotechnology and molecular biology has given rise to plant‐derived exosome‐like nanoparticles (PELNs) as a novel bioactive carrier. This development has garnered significant attention from the global medical community. As nanoscale vesicles released by cells, exosomes are widely present in animals and plants. They function as natural carriers capable of transporting diverse bioactive cargoes, encompassing functional proteins, lipid constituents, and genetic material (Y. Wang, Xiao, et al. 2023). In the context of current medical research, exosomes have become a focus of attention due to their crucial role in intercellular communication (Batrakova and Kim 2015; Krylova and Feng 2023). These extracellular vesicles are not only key participants in a variety of physiological processes but also significantly contribute to the pathogenesis of many diseases (Kok and Yu 2020; Rasihashemi et al. 2022).

PELNs, as extracellular vesicles derived from plant cells, exhibit remarkable structural and functional homology with their mammalian counterparts. They primarily consist of bioactive molecules such as lipids, proteins, nucleic acids, and secondary metabolites (Subha et al. 2023). Compared with animal‐derived exosomes, PELNs possess unique advantages, including their wide availability, superior stability, good biocompatibility, and lower immunogenicity. The aforementioned attributes render them optimal contenders for drug delivery systems and bestow upon them substantial potential for application in disease prevention and treatment (Karamanidou and Tsouknidas 2021; Madhan et al. 2024; Ou et al. 2023; Zheng et al. 2024).

Undoubtedly, PELNs are capable of modulating immune responses, alleviating inflammation, and facilitating tissue repair, which in turn leads to therapeutic outcomes. Moreover, they can function as drug delivery systems that accurately target diseased tissues, consequently improving drug bioavailability and therapeutic efficacy (Dad et al. 2021; Kathait et al. 2024; Ou et al. 2023). Meanwhile, they have demonstrated remarkable biological efficacy in the management of a range of diseases, such as tumors, infectious diseases, skin injury regeneration, and neurodegenerative disorders (Y. Cai, Zhang, et al. 2022; Teng et al. 2018; R. Wang, Zhang, et al. 2023). Up to now, PELNs have shown great potential in safeguarding crucial human organs such as the gastrointestinal tract, liver, skin, and brain (Anusha and Priya 2022; Feng et al. 2024; Kim, Zhu, et al. 2023; Subudhi et al. 2022; R. Wang, Zhang, et al. 2023). Specifically, ginger‐derived exosome‐like nanoparticles (GELNs) have been proven to have significant therapeutic effects, including anti‐inflammatory, anti‐tumor, antibacterial, and drug delivery capabilities (Anusha et al. 2023; X. Chen, Zhou, and Yu 2019; Kumar et al. 2022; L. Yan, Cao, et al. 2024; M. Zhang et al. 2016; H. Zhu and He 2023). Meanwhile, ginseng‐derived exosome‐like nanoparticles (GENs) have been shown to have cardioprotective effects (S. Yang, Guo, Chen, et al. 2025) and the ability to alleviate sepsis by loading miRNA (C. Ma et al. 2024). They also exhibit anti‐tumor, anti‐inflammatory, and antioxidant therapeutic effects (Cao et al. 2019; Han et al. 2022; Kim, Zhang, et al. 2023; Kim, Zhu, et al. 2023).

Although preliminary findings have highlighted the potential of PELNs in the context of disease prevention and treatment, there remains a necessity for more comprehensive research. This includes elucidating their precise mechanisms of action, establishing standardized extraction protocols, and conducting thorough assessments of their stability and safety. This study will undertake an exhaustive examination of the formation, extraction, and identification of PELNs. It will also explore their application advancements in different disease models. The objective is to establish a comprehensive theoretical framework and actionable guidelines to advance both fundamental research and clinical implementation. By systematically exploring the important roles of PELNs in disease prevention, diagnosis, and treatment, we aspire to provide novel concepts and pathways for the evolution of innovative nanomedicine approaches.

## 2 The Biogenesis of PELNs

**FIGURE 1:**
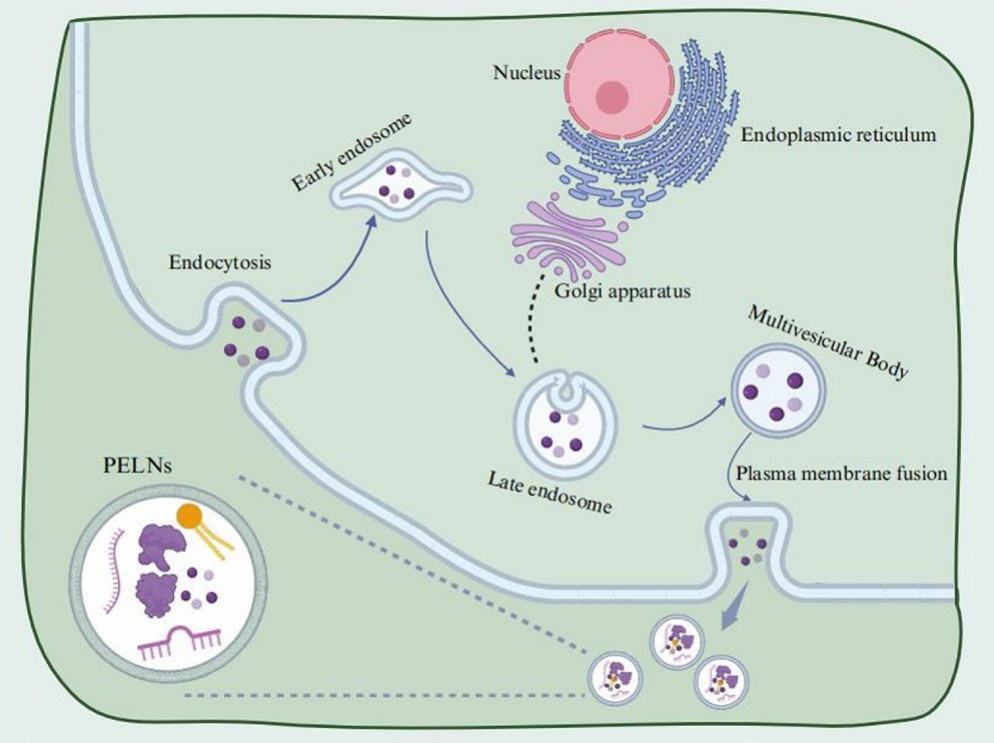
The biogenesis of PELNs‐MVBs pathway. PELNs formation is a critical cellular event. Endocytosis brings extracellular substances into early endosomes, which sort materials for recycling or degradation. In late endosomes, PELNs bind to membranes. The Golgi apparatus and endoplasmic reticulum contribute proteins and lipids to MVBs (multivesicular bodies), where PELNs are incorporated. MVBs, containing PELNs, can be directed to lysosomes or the plasma membrane, playing a role in cellular secretion. This pathway is vital for extracellular signaling and homeostasis. Created with BioRender.com.

PELNs have become a focus of considerable interest in recent research (Fan et al. 2022). They have not only demonstrated great potential in disease prevention and treatment but also provided novel approaches for drug delivery (Shao et al. 2023). A comprehensive grasp of their biogenesis is crucial for subsequent research and application. According to current research, the biogenesis of plant extracellular vesicles is believed to occur through three possible pathways: the EXosome POsitive (EXPO) organelle pathway, the multivesicular body (MVB) pathway, and the vacuolar pathway (B. Zhao et al. 2024). Among these, the MVB pathway is considered the primary route for the generation of PELNs. The initial step is the invagination of the plasma membrane, which forms early endosomes. These early endosomes then progress to maturity and connect with the trans‐Golgi network, leading to the development of MVBs (An et al. 2007). Intraluminal vesicles (ILVs) within MVBs have the capacity to encapsulate diverse substances such as RNA, DNA, and lipids. Upon the fusion of MVBs with the plasma membrane, ILVs are released into the extracellular space, which results in the formation of PELNs (Y. Li, Wang, et al. 2024; Wei et al. 2024). The MVB pathway is illustrated in Figure 1.

## 3 Isolation and Characterization

### 3.1 Isolation Methods of PELNs

Currently, the efficient enrichment of exosomes remains a significant challenge. With the advancement of scientific and technological methods, the extraction and isolation techniques for plant‐derived exosome‐like vesicles are continuously being refined. To achieve high recovery rates, high purity, and high‐throughput separation, researchers have employed a variety of extraction techniques tailored to different plant sources (Y. Liu et al. 2020). The most commonly used methods include ultracentrifugation, density gradient ultracentrifugation, and size‐exclusion chromatography (Z. Chen et al. 2024; L. Cui, Perini, et al. 2024; Lian et al. 2022).

Unlike the isolation methods for animal exosomes, the extraction of PELNs requires pre‐treatment of plant tissues prior to purification (B. Zhao et al. 2024) (Table 1). Plant tissues are first washed thoroughly and homogenized into juice. Coarse fibrous materials and larger particulates are removed from the juice by differential centrifugation at various speeds and durations (F. Wang, Li, et al. 2025). Subsequently, preliminary exosome fractions are acquired through the application of ultracentrifugation and sucrose density gradient centrifugation (Zhuang et al. 2015).

**TABLE 1:** Comparison of animal exosomes and PELNs.

Property	Animal exosomes	PELNs	References
Physical and chemical properties	Size: 30–200 nm, typically cup‐shaped or spherical	Size: 50–200 nm, diverse shapes	Y. Wang et al. (2022), Sergazy et al. (2025), Y.‐S. Chen, Lin, et al. (2019), Mun et al. (2025), Lei et al. (2025), Majewska et al. (2025)
Immunogenicity	Low immunogenicity when derived from the same species, but may cause immune reactions in xenotransplantation	Typically low immunogenicity, rarely causing immune reactions	
Production cost	Limited extraction sources (e.g., cell culture supernatant), high cost	Plant materials are widely available, low cost	
Yield	Low yield when extracted from cell culture, requiring a large number of cells and time	Large amount of plant tissue, relatively high yield	

In comparing the extraction methods for PELNs, it is important to note that each method has its own set of advantages and disadvantages (Table 2). Differential centrifugation is effective for removing larger particles but may not efficiently isolate the exosomes. Ultracentrifugation provides a more concentrated exosome fraction but can be time‐consuming and may result in contamination with other cellular debris. Sucrose density gradient centrifugation offers a higher degree of purification but requires careful handling and is more labor‐intensive.

**TABLE 2:** Comparison of extraction methods for PELNs.

Extraction method	Source	Advantages	Limitations	References
Differential centrifugation + ultracentrifugation	Brucea javanica	Retains intact miRNAs and lipids; reproducible	Equipment‐intensive; moderate yield	G. Yan, Xiao, et al. (2024)
Filtration (0.22 μm) + ultracentrifugation	Grape	Enables multi‐drug loading	Complex post‐modification	Farheen et al. (2024)
Differential ultracentrifugation + sucrose gradient	Mulberry bark	High purity; preserves vesicle integrity; scalable for large batches	Requiresexpensive ultracentrifuge; risk of vesicle aggregation at high g‐forces	Sriwastva et al. (2022)
Electrophoresis‐dialysis (ELD)	Lemon	No ultracentrifuge needed; scalable	Requires optimization of electric field	M. Yang et al. (2020)
PEG8000 precipitation	Green onion	Low cost, simple operation; scalable	Higher impurity levels; moderate purity	Yoon et al. (2024)
Ultrafiltration + size‐exclusion chromatography (SEC)	Cabbage	High purity; uniform particle size; suitable for drug loading	High equipment requirements; complex steps	You et al. (2021)
Hot water extraction + ExoEasy Maxi Kit	Atractylodes lancea rhizome	Retains miRNAs; suitable for dried herbs	Low yield; high impurity levels	Ishida et al. (2023)

**FIGURE 2:**
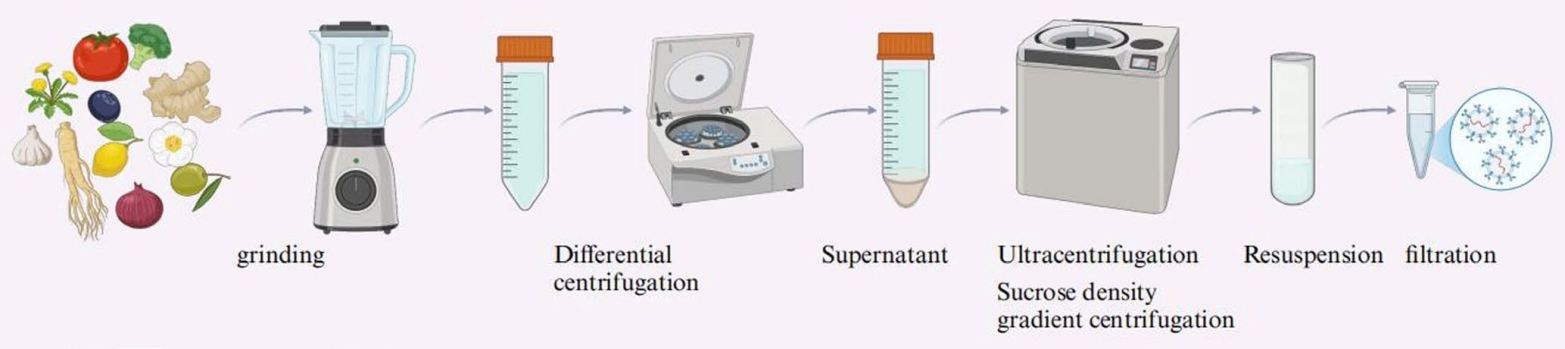
Sequential ultracentrifugation‐sucrose gradient for PELNs purification. The procedure commences with the homogenization of plant tissues to extract intracellular components. Subsequently, differential centrifugation is employed to isolate the supernatant enriched with PELNs. This is followed by ultracentrifugation through a sucrose density gradient to further purify the PELNs based on their density. The final step involves resuspension and filtration to concentrate the PELNs, ensuring a high degree of purity suitable for subsequent research endeavors. This methodical approach guarantees the efficient isolation and purification of PELNs. Created with BioRender.com.

Finally, the preliminary exosomes are dissolved in pre‐chilled phosphate‐buffered saline (PBS) and filtered to yield highly purified exosomes, which are stored at −80°C. This approach is now the predominant one for extracting PELNs. By integrating ultracentrifugation with sucrose density gradient centrifugation, the drawbacks of each method are mitigated, leading to the production of purer PELNs (see Figure 2).

### 3.2 Characterization Methods for PELNs

Analogous to mammalian exosomes, PELNs characterization necessitates multi‐modal analytical integration (Y. Liu et al. 2024). They are typically characterized through a combination of advanced analytical methodologies. Transmission electron microscopy serves as the primary technique for ultrastructural examination, providing nanoscale resolution that enables precise visualization of particle morphology and internal architecture. Concurrently, nanoparticle tracking analysis via dynamic light scattering provides quantitative data on size distribution and surface charge characteristics (Bai et al. 2024). Immunoblotting techniques further validate their molecular identity through the detection of signature membrane proteins, thereby establishing a robust framework for their biophysical and biochemical characterization. However, the specific protein markers for PELNs are not yet well defined (Suharta et al. 2021; K. Wang, He, et al. 2025), necessitating further research to identify them.

Moreover, we acknowledge the need for further discussion on the nanoparticle sizes listed in Table 3. Size variations can significantly impact therapeutic efficacy and bioavailability. Smaller nanoparticles enhance cellular uptake and tissue penetration, while larger ones offer greater stability and prolonged circulation (Lei et al. 2025; Majewska et al. 2025; B. Zhao et al. 2024).

**TABLE 3:** Isolation methods and sizes of PELNs from various plant sources.

Source	Isolation method	Size (nm)	References
Ginger	Ultracentrifugation	108.6 ± 2.60	Guo et al. (2025)
Ginseng	Sucrose density gradient ultra‐centrifugation	256	S. Yang et al. (2024)
Grapefruit	Sucrose density gradient ultra‐centrifugation	163.4	Huang et al. (2023)
Tea leaf	Sucrose density gradient ultra‐centrifugation	166.9	Q. Chen et al. (2023)
*Catharanthus roseus*	Sucrose buffer‐ultracentrifugation	141.7	Ou et al. (2023)
Mulberry bark	Sucrose density gradient ultra‐centrifugation	151.3 ± 45.4	Sriwastva et al. (2022)
Cabbage	Size‐exclusion chromatography	98.8	You et al. (2021)
*Citrus limon* L.	Sucrose density gradient ultra‐centrifugation	50–70	Raimondo et al. (2015)

In summary, the continuous improvement of extraction and characterization techniques for PELNs is crucial for advancing their application in biomedical research and therapeutic development. Future work should focus on identifying specific biomarkers for them and optimizing extraction protocols to enhance yield and purity. Additionally, elucidating the distinct characteristics of PELNs in comparison to animal‐derived exosomes could uncover innovative therapeutic approaches and solidify them as a viable option for nanomedical applications.

## 4 Exploring PELNs for Therapeutic Purposes

**FIGURE 3:**
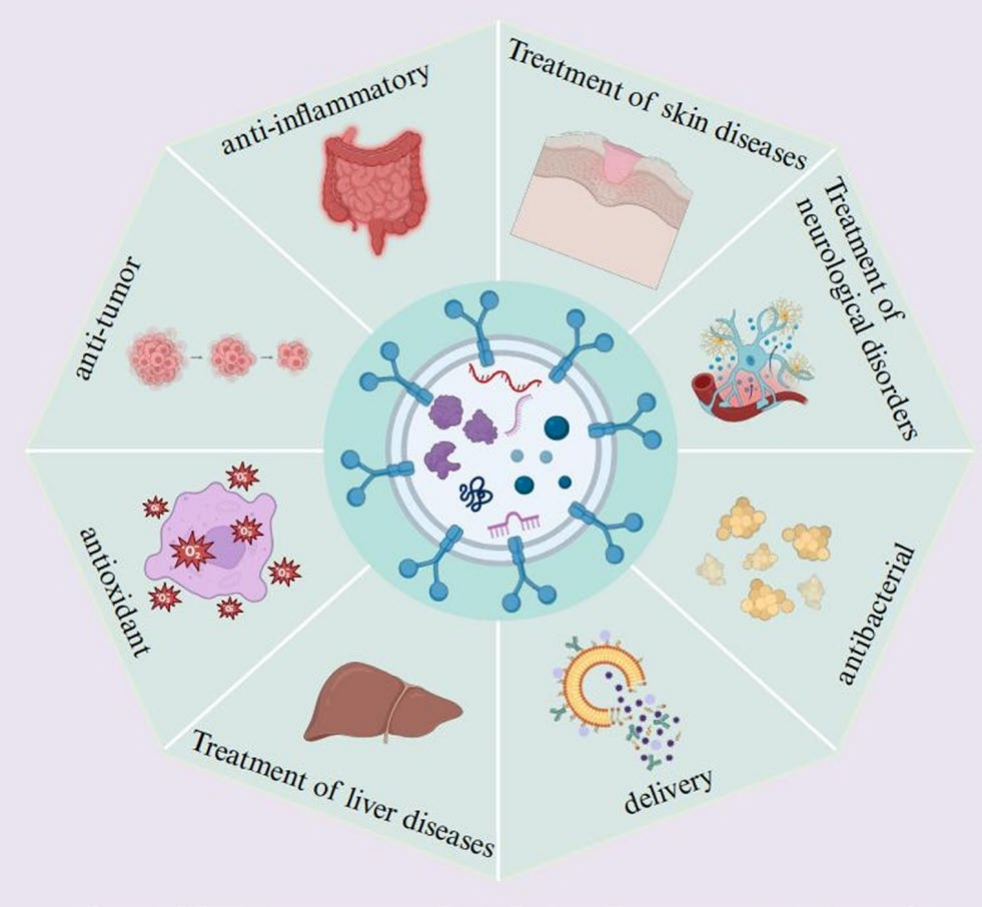
The potential of PELNs in therapeutic strategies. PELNs demonstrate considerable potential in therapeutic applications. They facilitate precise drug delivery to affected areas, including inflammation, dermatological conditions, cardiovascular diseases, liver disorders, and tumors, thereby enhancing treatment efficacy and minimizing side effects. This technology offers innovative approaches for managing a variety of illnesses. Created with BioRender.com.

PELNs have been proposed for the prevention or treatment of human diseases due to their broad availability and favorable biocompatibility. Despite the preliminary stage of current investigations regarding their therapeutic mechanisms, accumulating evidence substantiates their considerable clinical promise. This section primarily summarizes the multifunctional properties of PELNs (Figure 3).

### 4.1 Targeted Therapy for Inflammatory Diseases With PELNs

Increasing evidence suggests that PELNs exhibit significant anti‐inflammatory efficacy. Inflammation can be a primary cause of disease, and current anti‐inflammatory treatments often lead to a range of adverse effects. Therefore, researchers are focusing on finding naturally derived therapeutic agents.

Different research teams have found that honeysuckle, mulberry bark, garlic, ginseng, fresh rehmannia, and mugwort leaves have certain protective effects against colitis. The study by Li showed that honeysuckle MIR2911 can alleviate dextran sulfate sodium (DSS)‐induced colitis via precise modulation of the gastrointestinal microbiome, such as selectively suppressing pathogenic taxa while concurrently stimulating the proliferation of beneficial microbial species (W. Li, Ding, et al. 2024). Mukesh discovered that edible mulberry bark‐derived exosome‐like nanoparticles (MBELNs) alleviate experimental colitis in mice via HSPA8‐mediated activation of the AhR signaling cascade (Sriwastva et al. 2022). Garlic‐derived nanovesicles (GENs) deploy the abundant miRNA han‐miR3630‐5p to silence TLR4 mRNA, thereby abrogating MyD88‐mediated NF‐κB activation, attenuating IL‐6, TNF‐α, and related cytokines, and restoring epithelial tight‐junction integrity. Simultaneously, GENs reshape the microbiota—suppressing pro‐inflammatory taxa while enriching butyrate‐producing Lachnospiraceae—and the resulting short‐chain fatty acids further restrain NF‐κB (Z. Zhu et al. 2023). Kim reported that GENs have therapeutic effects on colitis by modulating the gut microbiota and immune microenvironment. Their anti‐inflammatory effects are achieved through the suppression of pro‐inflammatory cytokines, while their microbiota‐regulating activity restores microbial equilibrium and reinforces intestinal barrier function. These dual mechanisms synergistically ameliorate gastrointestinal inflammation and enhance overall digestive system performance (Kim, Zhang, et al. 2023). Qiu found that *Rehmanniae Radix* exosome‐like nanoparticles ferry miR‐7972, which silences GPR161 via 3′‐UTR targeting, derepresses the Hedgehog pathway (SHH/Ptch1 up, SMO/Gli1 down), and thereby quells LPS‐driven lung inflammation while rebalancing the gut microbiota (Qiu et al. 2023). Similarly, mugwort‐derived exosome‐like nanovesicles (FAELNs) exhibit therapeutic efficacy in the management of ulcerative colitis, through both cellular and animal model investigations demonstrating that FAELNs significantly reduce oxidative damage, decrease inflammatory cytokine production, and improve the inflammatory microenvironment, while also alleviating intestinal barrier damage and modulating gut microbiota diversity (Y. Li et al. 2025).

Although all cited investigations relied on DSS to precipitate acute colitis in mice, inter‐study heterogeneity in DSS concentration and exposure length generated markedly disparate baseline inflammatory intensities, thereby confounding the appraisal of therapeutic efficacy. Moreover, the acute, self‐limiting phenotype induced by DSS diverges fundamentally from the chronic, relapsing nature of human ulcerative colitis in both immunological milieu and microbial architecture; consequently, the extant pre‐clinical findings provide an insufficient foundation for prognosticating translational success.

**FIGURE 4:**
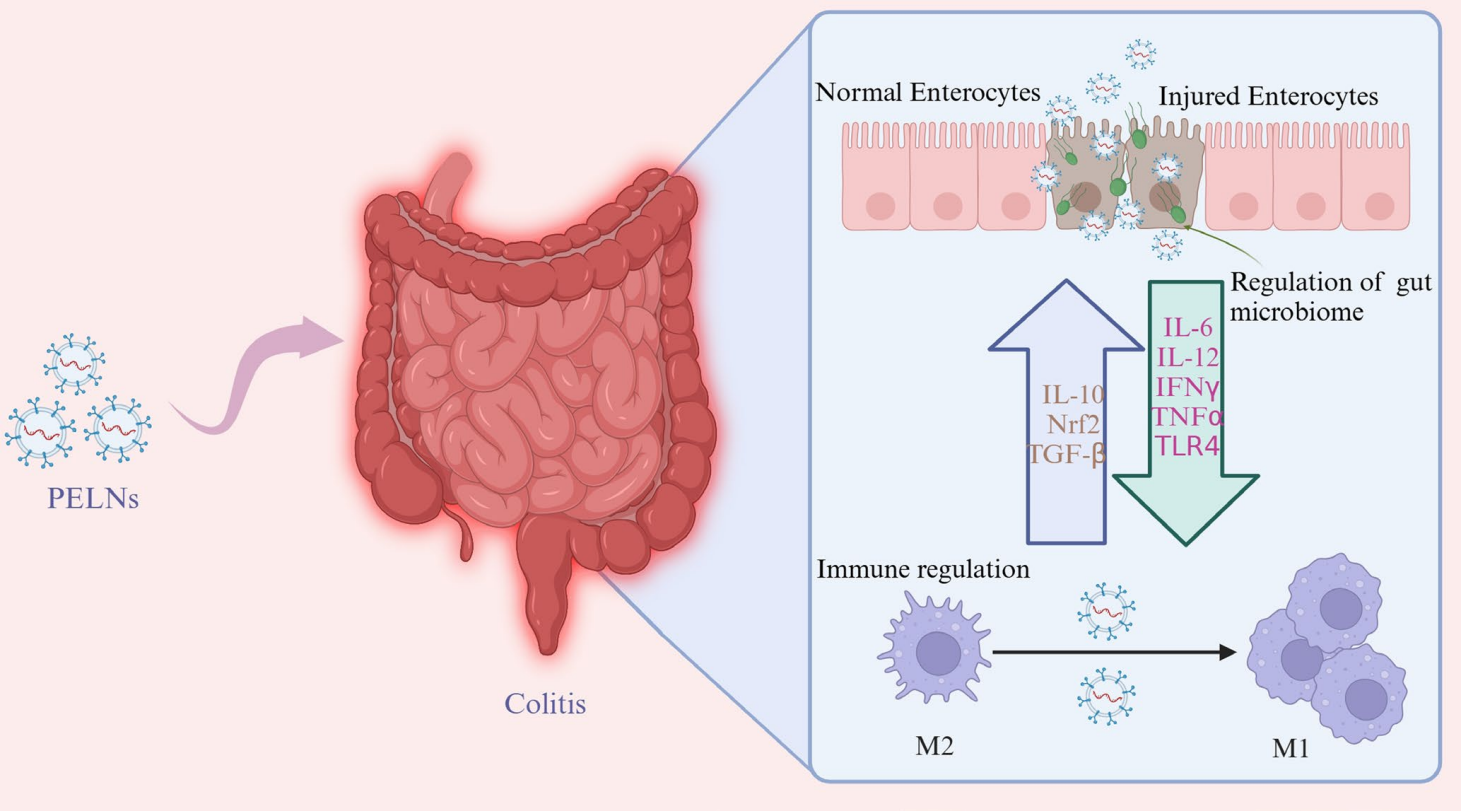
The effects of PELNs on colitis. During colitis, the intestinal epithelial barrier is weakened, leading to increased permeability and inflammation. PELNs aid in restoring the integrity of the intestinal epithelium by promoting the recovery of healthy enterocytes and mitigating the damage from injured ones. PELNs also play a key role in regulating the gut microbiome, influencing the production of various cytokines and growth factors such as IL‐6, IL‐12, IFN‐γ, TNF‐α, and TGF‐β, which are central mediators in the inflammatory response. By modulating these factors, PELNs contribute to a balanced immune response and help resolve inflammation. Additionally, PELNs are involved in immune regulation, facilitating the shift of macrophages from a pro‐inflammatory M1 phenotype to an anti‐inflammatory M2 phenotype, which helps reduce inflammation and promote tissue repair. Created with BioRender.com.

The findings highlight the diverse anti‐inflammatory mechanisms of PELNs, which encompass the modulation of gut microbiota, immune regulation, and targeting of specific signaling pathways. By exerting anti‐inflammatory effects while potentially circumventing the adverse reactions associated with conventional therapies, they emerge as a promising candidate for next‐generation therapeutic agents (Figure 4).

### 4.2 Novel Applications of PELNs in Cancer Therapy

Recent investigations into exosome‐like nanoparticles derived from edible fruits and vegetables have demonstrated significant therapeutic potential. These plant‐derived nanoparticles frequently display comparable or enhanced bioactivity relative to their source materials. Given these promising findings, researchers hypothesize that PELNs may enhance the treatment efficacy of refractory diseases, such as cancer. An expanding corpus of scientific evidence increasingly substantiates the tumor‐suppressive properties of PELNs. These results highlight the therapeutic promise of PELNs as innovative candidates for managing malignancies and other complex pathologies (L. Cui, Perini, et al. 2024; H. Liu et al. 2023) (Table 4).

**TABLE 4:** Tumors suppressed or alleviated by plant‐derived exosome‐like nanoparticles.

Plant	Cell line	Animal model	Tumor	References
Lemon juice	The human lung carcinoma cell line A549, the chronic myeloid leukemia cell line LAMA84 cells	Male NOD/SCID mice	Chronic myeloid leukemia (CML)	Raimondo et al. (2015)
Ginseng	The murine melanoma cell line (B16F10), breast cancer cell line (4T1) and human embryonic kidney cell line (HEK293T)	C57BL/6 mice	Melanoma	Cao et al. (2019)
Lemon	The gastric cancer cell line AGS, BGC‐823, and SGC‐7901	Female BALB/c nude mice	Gastric cancer	M. Yang et al. (2020)
Asparagus cochinchinensis	Human HCC cell lines (Hep G2, SMMC‐7721, and Hep 3B)	BALB/c mice	Hepatocellular carcinoma	L. Zhang et al. (2021)
Tea leaf	The mouse colon carcinoma cell line (CT‐26), human breast cancer cell line (MCF‐7 cell) and mouse breast cancer cell line (4T1)	Female BALB/c nude mice	Breast tumor	Q. Chen et al. (2023)
Brucea javanica	4T1 cells and HUVECs	BALB/c mice	Breast tumor	G. Yan, Xiao, et al. (2024)
Ginger	C57BL/6 murine melanoma B16F10 cells	C57BL/6 J mice	Melanoma	Teng et al. (2025)
Ginger	The mouse 4T1 breast tumor cell line	Female BALB/c mice	Breast tumor	Guo et al. (2025)
Radix Polygoni Multiflori	The human LC cell line (SMMC‐7721)	—	Liver cancer	M. Yang, Xu, and Wang (2025)
*Platycodon grandiflorum*	4T1 cells, A549 cells and Raw 264.7 cells	Balb/C nude mice	Breast tumor	M. Yang, Guo, Li, et al. (2025)

Tea leaf‐derived natural nanovesicles are abundant in diverse bioactive compounds. Experimental evidence indicates that administration of them significantly attenuates murine colonic adenocarcinoma development while concurrently suppressing Ki67 expression in neoplastic tissues. Additionally, this therapeutic intervention significantly enhanced IL‐10 expression while concurrently suppressing pro‐inflammatory cytokine production. These data demonstrate that nanovesicles isolated from tea leaves possess potent anti‐inflammatory properties and effectively suppress the advancement of colorectal neoplasms (Zu et al. 2021). Moreover, the research team identified that exosome‐like nanovesicles from fresh tea leaves (TLNTs) exhibit pronounced antiproliferative activity against breast cancer cells, establishing their viability as a candidate therapeutic modality for this malignancy (Q. Chen et al. 2023). In 2022, the team successfully isolated a substantial quantity of exosome‐like nanovesicles (TFENs) from edible tea flowers for the first time. TFENs demonstrated significant cytotoxicity toward malignant cells through ROS induction while preserving excellent biocompatibility with non‐tumorigenic cells (Q. Chen et al. 2022). Animal studies revealed that intravenous or oral administration of TFENs led to their accumulation in breast tumors and lung metastases, effectively inhibiting tumor growth and metastasis (Q. Chen et al. 2022). Across the cited investigations, animals received PELNs either intragastrically or via the tail vein; however, the administered doses ranged four‐fold (50–200 mg kg^−1^) and no study determined the resultant vesicular concentrations in plasma or neoplastic tissue. Consequently, dose–response relationships remain incomparable among datasets. Additionally, brucea javanica exosome‐like nanovesicles (BF‐Exos) deliver 10 bioactive miRNAs across species barriers into 4T1 TNBC cells, where they silence the PI3K/Akt/mTOR axis by suppressing p‐PI3K, blocking PIP2‐to‐PIP3 conversion, and deactivating downstream p‐Akt and p‐mTOR. This cascade unleashes mitochondrial apoptosis via sequential Caspase‐9/3 activation. Orthotopic 4T1 xenografts receiving intratumoral BF‐Exos exhibited marked tumor regression without systemic toxicity, validating a safe and potent plant‐derived miRNA nanoplatform for TNBC therapy (G. Yan, Xiao, et al. 2024). Furthermore, selenium‐enriched broccoli‐derived exosome‐like nanovesicles (ELNs) have shown superior anti‐pancreatic cancer effects compared to traditional broccoli‐derived ELNs (X. Wang, Wu, et al. 2023).

These investigations collectively highlight the broad‐spectrum anticancer efficacy and adaptability of PELNs, demonstrating their value as multi‐target therapeutic platforms for diverse malignancies. Characterized by their capacity to modulate key signaling pathways, augment immune responses, and selectively target cancer cells, ELNs offer a compelling alternative for future cancer therapies. Their favorable profile of low immunogenicity and high biocompatibility further enhances their suitability as a next‐generation treatment modality.

### 4.3 The Unique Role of PELNs in Antioxidative Stress

In contrast to animal‐derived exosomes, plants are abundant in polyphenols and flavonoids, which exhibit remarkable capacities for scavenging free radicals (Fernando et al. 2019; P. Ma and Wang 2023). These bioactive compounds are capable of effectively mitigating cellular damage induced by oxidative stress (Hossain et al. 2022).

Strawberry‐derived exosome‐like nanoparticles (SELNs) confer concentration‐dependent cytoprotection to human mesenchymal stromal cells (MSCs) by attenuating oxidative damage (Perut et al. 2021). When broccoli‐derived vesicles (BDVs) are applied to cells exposed to H_2_O_2_ or 6‐HODA, they significantly reduce levels of reactive oxygen species (ROS) (Hossain et al. 2022). Ginseng root‐derived exosome‐like nanoparticles (GrDENs) protect cells from UVB‐induced oxidative damage through antioxidant effects and exhibit anti‐inflammatory and anti‐aging activities by inhibiting AP‐1 signaling under H_2_O_2_‐induced oxidative stress (Choi et al. 2024). Blueberry‐derived exosome‐like nanoparticles (BELNs) mitigate oxidative damage in both rotenone‐challenged HepG2 cells and high‐fat diet‐fed C57BL/6 mice by mitigating oxidative stress while restoring the mitochondrial electrochemical gradient (W. Zhao et al. 2022). *Centella asiatica* ‐derived vesicles, rich in polyphenols, demonstrate robust free radical scavenging and metal ion chelation abilities, effectively reducing intracellular ROS levels induced by hydrogen peroxide (Chang et al. 2024).

In conclusion, the collective findings emphasize the substantial potential of PELNs as natural antioxidants and agents capable of mitigating oxidative stress. PELNs, which naturally concentrate bioactive phytochemicals such as polyphenols and flavonoids, have been extracted from diverse botanical matrices, including strawberries, broccoli, ginseng, blueberries, and *Centella asiatica* . These nanoparticles have demonstrated exceptional abilities to scavenge free radicals and attenuate oxidative stress by diminishing cellular ROS concentration, and safeguard cells against oxidative damage. These attributes position them as valuable candidates for the advancement of innovative therapeutic modalities targeting diseases associated with oxidative stress.

### 4.4 Treatment of Liver Diseases

Exosome‐like nanovesicles derived from fresh mulberry leaves (MLNPs) are rich in functional lipids, proteins, and flavonoids, demonstrating significant liver targeting and enrichment capabilities. These properties enable effective inhibition of liver tumor growth and modulation of gut microbiota balance (Gao et al. 2024). Garlic‐derived exosome‐like nanovesicles (GaELNVs) mitigate hepatic injury through a multi‐target mechanism: suppression of pro‐inflammatory mediator release, inhibition of apoptosis and inflammasome activation, and stimulation of autophagic flux. This integrated cellular response collectively confers hepatoprotective efficacy (X. Zhao et al. 2023). Asparagus cochinchinensis‐derived vesicles (ACNVs) demonstrate concentration‐dependent suppression of hepatocellular carcinoma growth while simultaneously triggering programmed cell death (L. Zhang et al. 2021). Tangerine peel‐derived exosome‐like nanovesicles (TNVs) improve liver function by reducing hepatic lipogenic factors and bile acid accumulation, lowering blood lipids, and reducing hepatic lipid accumulation (Zou et al. 2024). Contemporary investigations have revealed that the exosome‐like nanoparticles derived from *Polygonum multiflorum* (RPM‐ELNs) are efficiently internalized by hepatocellular carcinoma cells, thereby effectively inhibiting their proliferation and migration in vitro (M. Yang, Xu, and Wang 2025).

In conclusion, the findings collectively underscore the therapeutic potential of plant‐derived exosome‐like nanovesicles in targeting hepatic diseases and modulating the associated pathological cascades. Their natural origin and abundance of bioactive constituents render them compelling candidates for the development of innovative liver‐targeted therapeutic strategies.

### 4.5 Treatment of Skin Diseases

As the body's largest organ, the skin functions as a vital protective barrier against environmental insults and pathogenic invasion (Abels and Angelova‐Fischer 2018). PELNs serve as pivotal regulators in cutaneous homeostasis and disease progression, functioning as critical orchestrators of dermal physiology. They have the potential to become novel therapeutic options for the repair, regeneration, and rejuvenation of skin tissue (Di Raimo et al. 2024).

Tomato‐derived nanovesicles enhance the motility of keratinocytes and fibroblasts, thereby expediting re‐epithelialization and extracellular matrix deposition during wound repair. This capacity to stimulate key cellular components of the healing cascade suggests translational relevance for refractory and chronic cutaneous ulcers (Daniello et al. 2024). In contrast to tomato‐derived nanovesicles, wheat‐derived nanovesicles not only significantly stimulate the motility and division of endothelial cells, keratinocytes, and dermal fibroblasts, thereby accelerating the orchestrated cellular events that drive efficient cutaneous repair (Şahin et al. 2019). *Centella asiatica* ‐derived vesicles exhibit significant biological functions, effectively reducing intracellular melanin production and tyrosinase activity, while upregulating barrier‐associated genes including type I procollagen, aquaporin 3, and filaggrin (Chang et al. 2024). Moreover, the exosome‐like nanoparticles derived from **Ecklonia cava** have been demonstrated to enhance the skin's antioxidant capacity, promote collagen synthesis, and expedite skin regeneration, thereby providing a robust scientific foundation as innovative therapeutic modalities for age‐related skin rejuvenation (Batsukh et al. 2024).

In addition, studies have demonstrated that PELNs may exhibit enhanced therapeutic effects when combined with other substances. A hydrogel‐embedded delivery system for olive leaf‐derived exosome‐like nanovesicles confers dual benefits by attenuating UV‐induced photodamage while simultaneously stimulating cutaneous repair and regeneration (Z. Wang, Yuan, et al. 2024). Huang developed a novel multifunctional nanovesicle as therapeutic candidates for autoimmune dermatoses. This nanovesicle is composed of grapefruit‐derived exosome‐like nanovesicles (GEV) combined with the immunosuppressive agent CX‐5461 and further fused with CCR6+ nanovesicles. This formulation can better target inflamed tissues, effectively reduce inflammatory cytokines, inhibit Th17 cells, and promote Treg cell infiltration, exhibiting marked efficacy in preclinical murine models of psoriatic and atopic dermatitis (Huang et al. 2023).

Taken together, these findings underscore the extensive clinical promise of PELNs in managing diverse skin conditions, spanning from chronic wounds and UV‐induced damage to autoimmune and inflammatory dermatoses. The natural composition of PELNs, coupled with their capacity to engage with cellular signaling networks and modulate immune activity, renders them attractive prospects for advancing innovative skin treatment modalities.

### 4.6 Potential Applications of PELNs in Neurological Diseases

PELNs have exhibited the capacity to penetrate the blood–brain barrier (BBB) through receptor‐guided transcytosis and lipid bilayer fusion pathways. This characteristic establishes a promising strategy for both detecting and managing central nervous system pathologies (H. Cai, Huang, et al. 2022; R. Wang, Zhang, et al. 2023; W. Wang, Su, et al. 2024).

Engineered *Pueraria lobata* exosomes (Pu‐Exos‐PR) facilitate efficient brain enrichment through intranasal delivery, transporting bioactive traditional Chinese medicine miRNAs. This therapeutic intervention demonstrates marked neuroprotective efficacy in Parkinson's disease by preserving dopaminergic neuron viability, attenuating oxidative injury, and ameliorating both motor dysfunction and non‐motor manifestations of the disease (Y. Xu 2024). Exosome‐like nanoparticles derived from Lycium ruthenicum (LRM‐ELNs) significantly reduce Aβ‐induced oxidative stress in HT22 cells by activating the Nrf2/HO‐1/NQO1 signaling pathway, suggesting their potential for Alzheimer's disease (AD) therapy (Y. Zhang et al. 2024). Additionally, exosome‐like nanoparticles from *Momordica charantia* (ELNs) have been shown to reduce BBB damage caused by ischemia–reperfusion and inhibit neuronal programmed cell death via the AKT/GSK3βsignaling pathway (H. Cai, Huang, et al. 2022). Research indicates that garlic‐derived exosome‐like nanoparticles (GENs) enhance targeting of the BBB and glioblastoma, emerging as a glioblastoma treatment in both cellular and animal models while significantly inhibiting glioblastoma progression (Kim, Zhu, et al. 2023). Engineering GELNs (Exo@tac) has been demonstrated to improve outcomes in Parkinson's disease (W. Cui, Guo, et al. 2024).

In conclusion, the collective findings emphasize the innovative and effective potential of PELNs as a transformative approach for the diagnosis and treatment of neurological disorders. They sourced from diverse botanical origins exhibit BBB permeability through receptor‐guided transcytosis and lipid bilayer fusion pathways. This trans‐endothelial transport capability enables them to deliver bioactive cargoes into the central nervous system, establishing their potential as nanocarriers for neurological therapeutic. This ability enables targeted drug delivery to the central nervous system, positioning PELNs as a revolutionary platform within the field of neurotherapeutics.

### 4.7 The Unique Role of PELNs in Antimicrobial Infections

Bacterial infections pose a persistent threat to global public health. With the continuous emergence of antibiotic‐resistant strains, the need for innovative non‐antibiotic therapeutic approaches becomes increasingly urgent (Spellberg et al. 2013; Y. Wang, Guo, et al. 2024).

Research has demonstrated that dandelion‐derived exosome‐like nanovesicles exhibit significant antitoxin activity by specifically binding to the exotoxins of *Staphylococcus aureus* ( *S. aureus* ), thereby effectively protecting host cells. They show strong binding affinity and high stability, neutralizing the exotoxins' toxicity in in vivo experiments (Tan et al. 2024). Ginger‐derived exosomes possess notable neutralizing capabilities, effectively counteracting the bacterial toxins secreted by *S. aureus* , thus reducing damage to normal tissues (Qiao et al. 2022; Y. Wang, Guo, et al. 2024). Additionally, GELNs are selectively absorbed by the oral pathogenic bacterium *Porphyromonas gingivalis* through a phosphatidic acid (PA)‐dependent interaction with the heme‐binding protein 35 (HBP35) on the bacterial surface (Sundaram et al. 2019).

Collectively, these findings underscore the potential of PELNs as an innovative non‐antibiotic therapeutic modality, offering a novel approach to combat bacterial infections while mitigating the risk of antibiotic resistance development.

### 4.8 PELNs as a Platform for Delivering Bioactive Molecules

PELNs hold great promise as natural or engineered drug delivery systems (Cong et al. 2022; R. Wang, Zhang, et al. 2023; Yi et al. 2025), capable of transporting a diverse array of bioactive and chemical molecules, thereby effectively enhancing inter‐species communication (B. Zhao et al. 2024). Li demonstrated that while naked MIR2911 can penetrate gut bacteria, it does not significantly modulate the growth of *Escherichia coli* . In contrast, MIR2911 encapsulated within seviroxin exerts significant regulatory effects on these bacteria by targeting the dnaG gene (W. Li, Ding, et al. 2024). Exosome‐like nanoparticles derived from Brucea javanica (BF‐Exos) have been identified as an efficient nanoplatform for delivering active miRNAs, inhibiting breast cancer growth, metastasis, and neovascularization through the PI3K/Akt/mTOR signaling cascade (G. Yan, Xiao, et al. 2024). siRNA‐loaded GELNs significantly inhibited tumor growth in xenograft models when administered intravenously (Z. Li et al. 2018). GELNs have been proven to be a promising nanoplatform for delivering plant‐derived miRNAs to mammalian stem cells, promoting neurodifferentiation across cellular and animal models (X.‐H. Xu et al. 2021). Emerging evidence indicates that exosome‐like nanoparticles derived from ginger and loaded with indocyanine green (GDNPs@ICG) exhibit potent anti‐tumor effects. Specifically, they significantly reduce angiogenesis in breast tumors, inhibit metastatic progression, activate robust anti‐tumor immune responses, and induce cellular senescence (Guo et al. 2025). Additionally, studies have shown that grapefruit‐derived exosome‐like nanoparticles can load the exogenous protein HSP70 and deliver it to GL‐Tr cells (Kilasoniya et al. 2023).

Current in vivo evaluations have relied predominantly on intravenous or enteral administration, yet the long‐term immunobiology of plant‐derived vesicles remains largely uncharted. Their rich lipid bilayer and surface glycoproteins render them potentially immunogenic, capable of complement activation or eliciting anti‐vector antibodies; however, published protocols seldom exceed 4 weeks, precluding appraisal of chronic immune surveillance, memory responses, or accelerated clearance. Consequently, the translational dossier is undermined by sparse data on payload quantification, biodistribution fidelity, systemic tolerability, and model fidelity. Establishing harmonized metrics for encapsulation efficiency, coupled with comprehensive multi‐organ pharmacokinetics, extended immunogenicity surveillance, and pre‐clinical systems that more faithfully recapitulate human oncologic or neurologic pathophysiology, is therefore imperative to advance PELNs from proof‐of‐concept to clinically viable therapeutics.

To summarize, PELNs have surfaced as highly adaptable and efficient platforms for drug delivery, demonstrating the ability to transport diverse bioactive and chemical entities across different species. These insights underscore their capacity them to facilitate communication between species and influence biological functions in both microbial and mammalian contexts.

To date, a significant body of research has demonstrated the substantial therapeutic potential of PELNs in disease treatment. However, the pharmacokinetics and systemic distribution of PELNs have not been extensively studied. They are influenced by various factors, including their source and route of administration. Further research is needed to elucidate the pharmacokinetic behavior of PELNs under different administration routes to optimize their clinical applications.

## 5 Conclusion

PELNs constitute a distinct class of extracellular vesicles originating from plant cells, sharing significant architectural and functional similarities with mammalian exosomes. These nanoscale carriers, naturally released through cellular secretion pathways, offer unique botanical advantages for therapeutic applications. Composed primarily of lipids, nucleic acids, proteins, and secondary metabolites, PELNs present multiple therapeutic advantages compared to mammalian exosomes. These include broader availability, lower cost, higher biosafety, enhanced stability, and reduced immunogenicity. These attributes position PELNs as a valuable tool in personalized and precision medicine, with broad applications in the treatment of various diseases.

Botanically derived exosome‐mimetic nanovesicles manifest a broad pharmacological profile encompassing oncological suppression, oxidative damage prevention, and immune modulation, establishing their candidacy for clinical applications in neoplastic, inflammatory, and dermatological conditions. The therapeutic versatility of PELNs originates from the integrated functionality of their constituent biomolecules—membrane lipids, genetic material, protein cargo, and phytochemicals—which collectively modulate intracellular signaling cascades, redox homeostasis, and immunological responses. This multi‐target regulatory capacity distinguishes them as a distinctive class of plant‐based nanotherapeutics capable of simultaneously addressing multiple pathophysiological pathways in complex disease states. Engineering modifications have significantly enhanced the targeting specificity and bioavailability of them, thereby augmenting their therapeutic efficacy. However, the stability and functionality of miRNAs and proteins during storage and delivery remain largely unexplored, which is critical for maintaining therapeutic efficacy. Future research must delve into the stability and functionality of these biomolecules during storage and delivery to ensure their effectiveness and safety in clinical applications.

Despite encouraging preclinical data, research on PELNs is still in its infancy compared to mammalian exosomes, with several challenges to overcome for clinical application. Methodological heterogeneity and mechanistic opacity limit progress. The lack of standardized isolation and purification schemes compromises batch‐to‐batch consistency, hindering reproducibility and scalable manufacture. The molecular mechanisms governing their bioactivity, including uptake routes and downstream signaling in mammalian cells, remain largely unknown. Rigorous toxicological appraisal, including immunogenicity profiling and chronic exposure assessment, is essential for regulatory‐compliant preclinical dossiers. A thorough investigation of these aspects is prerequisite to advancing PELNs from the laboratory to clinical settings.

Future efforts must shift from protocol optimization to mechanistic dissection and therapeutic context re‐engineering. Integrating single‐vesicle‐resolution mass spectrometry with spatial transcriptomics will generate high‐resolution spatiotemporal maps of PELNs within mammalian hosts, identifying interspecies signaling hubs. Machine‐learning‐guided molecular fingerprinting will enable the design of programmable membrane interfaces that release cascade drugs in inflammatory microenvironments. Parallel operation of an organoid‐based microphysiological immune‐profiling platform will predict patient‐specific immunological liabilities and streamline regulatory processes. Additionally, addressing the translational challenges and regulatory pathways for plant‐derived nanomedicines is crucial. Overcoming challenges such as scalable production, safety and toxicity assessments, and therapeutic stability is essential for clinical translation. Compliance with regulatory guidelines from agencies like the FDA and EMA, which involve preclinical studies, clinical trial design, adverse event reporting, and post‐marketing surveillance, is necessary for the clinical application of plant‐derived nanomedicines.

To encapsulate, PELNs constitute a promising therapeutic platform for augmenting the body's antioxidant defenses and treating a variety of diseases. Addressing current challenges, including the stability and functionality of cargo during storage and delivery, as well as translational and regulatory hurdles, will be crucial for their successful clinical application.

## Author Contributions

Zhaofeng Liang, Jiajia Jiang, and Wenrong Xu designed the research. Zihan Gao, Wenhui Yang, Yuning Gu, and Wenrong Xu analyzed data. Zihan Gao, Zhaofeng Liang, and Jiajia Jiang contributed to the writing and revisions. Data authentication is not applicable. All authors have read and approved the final version of the manuscript.

## Ethics Statement

The authors have nothing to report.

## Consent

The authors have nothing to report.

## Conflicts of Interest

The authors declare no conflicts of interest.

## Data Availability

Data sharing not applicable to this article as no datasets were generated or analyzed during the current study.
